# Clinical Effects of Form-Based Management of Forceps Delivery under Intelligent Medical Model

**DOI:** 10.1155/2021/9947255

**Published:** 2021-05-31

**Authors:** Siming Xin, Zhizhong Wang, Hua Lai, Lingzhi Liu, Ting Shen, Fangping Xu, Xiaoming Zeng, Jiusheng Zheng

**Affiliations:** Department of Obstetrics, Maternal and Child Health Hospital Affiliated to Nanchang University, Nanchang 330006, Jiangxi, China

## Abstract

**Background:**

Forceps delivery is one of the most important measures to facilitate vaginal delivery. It can reduce the rate of first cesarean delivery. Frustratingly, adverse maternal and neonatal outcomes associated with forceps delivery have been frequently reported in recent years. There are two major reasons: one is that the abilities of doctors and midwives in forceps delivery vary from hospital to hospital and the other one is lack of regulations in the management of forceps delivery. In order to improve the success rate of forceps delivery and reduce the incidence of maternal and neonatal complications, we applied form-based management to forceps delivery under an intelligent medical model. The aim of this work is to explore the clinical effects of form-based management of forceps delivery.

**Methods:**

Patients with forceps delivery in Maternal and Child Health Hospital Affiliated to Nanchang University were divided into two groups: form-based patients from January 1, 2019, to December 31, 2020, were selected as the study group, while traditional protocol patients from January 1, 2017, to December 31, 2018, were chosen as the control group. Then, we compared the maternal and neonatal outcomes of these two groups.

**Results:**

There were significant differences in the maternal and neonatal adverse outcomes such as rate of postpartum hemorrhage, degree of perineal laceration, and incidence of neonatal facial skin abrasions between the two groups, whereas differences in the incidence of asphyxia and intracranial hemorrhage were not significant.

**Conclusions:**

Form-based management could help us assess the security of forceps delivery comprehensively, as it could not only improve the success rate of the one-time forceps traction scheme but also reduce the incidence of maternal and neonatal adverse outcomes effectively.

## 1. Introduction

During the information era, medical and health field was gradually moving towards informationization and intellectualization. The intelligent medicine was a cross-discipline on the integration of life science and information technology, and it was a new stage of information construction in the healthcare field. By using information management, patients' clinical data could be fully recorded and tell us more about the situation of patients, which could help us to design effective treatments.

Under the environment of high cesarean section rate, we have the responsibility to promote vaginal birth and reduce primary cesarean birth rate [1, 2]. In order to obtain this goal, we need to put intelligent medicine into our daily medical works for reducing the morbidity of perinatal pregnant women and newborns. Through this way, we could reduce the evaluation errors caused by differences in clinical experience and other factors, so as to reduce the incidence of perinatal pregnant women and newborns [3]. In recent years, intelligent medicine has been widely used in prenatal fetal heart monitoring [4, 5], vaginal trial delivery model prediction after cesarean section [6], prediction model of vaginal birth after cesarean, and premature delivery [7]. It also has been used in postpartum hemorrhage prediction [8, 9]. During the time, it has achieved fairly good results in vaginal midwifery training [10].

Vaginal forceps delivery, one of the surgical vaginal methods, could resolve cephalic dystocia effectively, for example, maternal exhaustion, maternal cardiac disease and a need to avoid pushing in the second stage of labor, prolonged second stage of labor, and nonreassuring fetal heart rate patterns in the second stage of labor [11]. Under these conditions, forceps delivery could be accomplished more safely and quickly than cesarean. Therefore, the skill of forceps delivery was one of the most important clinical skills required for obstetricians and midwives [12]. Of course, forceps delivery not only requires the above skill but also needs accurate judgment and systematic evaluation of the patient's condition in advance. In recent decades, severe laceration of the birth canal, postpartum hemorrhage, and neonatal injury caused by forceps delivery have been constantly reported [13, 14]. Our hospital began the construction of an intelligent hospital in 2018 and has successively introduced information systems such as integrated platform, HIS, LIS, and HRP. Relying on these information platforms, we were able to implement form-based management of forceps delivery since January 1, 2019. In order to explore the effectiveness of form-based management of forceps delivery in improving the success rate of forceps delivery and reducing the incidence of adverse maternal and fetal outcomes, we were reviewing and analyzing the maternal and infant outcomes of patients with forceps delivery in our hospital during the period from January 1, 2017, to December 31, 2020.

## 2. Materials and Methods

### 2.1. Study Population

This retrospective cohort study was conducted at the Maternal and Child Health Hospital Affiliated to Nanchang University. The study population consisted of pregnant women who underwent forceps delivery from January 1, 2017, to December 31, 2020. Cases with forceps delivery managed by form-based management from January 1, 2019, to December 31, 2020, were established as the study group, and cases with forceps delivery managed by traditional protocol from January 1, 2017, to December 31, 2018, were established as the control group. The inclusion criteria were as follows: single full-term fetus, fetal position being cephalic position, indications for forceps delivery being prolonged second stage of labor or suspicion of immediate fetal distress or maternal complications, and low forceps with Kielland or Simpson forceps. The position of the fetal head in low forceps was that the lowest point of fetal cranial mass was located at or below +3 cm but it did not reach the pelvic floor. The exclusion criteria were huge babies and scarred uterus. The labor process in this study adopted new labor process standards [15]. Eligible women were identified from the hospital data management system, and all study participants were informed of the risks associated with forceps delivery and signed an informed consent form before undergoing forceps delivery.

### 2.2. Clinical Data Collection

Our hospital has introduced a medical record information system and medical record management system. Information between these two systems was completely interoperable. All medical records in this study were retrieved through the medical record management system. The retrieval strategy was: low forceps delivery as the procedure code, procedure time of January 1, 2019, to December 31, 2020, for the study group and January 1, 2017, to December 31, 2018, for the control group, the discharge code was singleton live birth. The delivery data was entered into the hospital data management system immediately after delivery by the midwives responsible for the ongoing care of the woman. Maternal data included age, height, weight, gravidity, parity, gestational age, indication of forceps delivery, instrument used, estimated blood loss, and degree of perineal laceration. Newborn data included birth weight, Apgar scores, umbilical artery blood pH value, intracranial hemorrhage, and facial skin injury.

## 3. Form-Based Forceps Delivery Management Program

From January 1, 2019, to December 31, 2020, we used form-based management of vaginal forceps delivery including a delivery room safe delivery verification form (Table 1) and a forceps delivery verification form (Table 2), which was verified by senior physicians, resident physicians, and midwives to systematically standardize forceps operations with the following steps. In particular, the senior doctors and midwives who were allowed to participate in this study should have the following qualifications: senior doctors should have intermediate or above titles, have at least 2 years of work experience in the delivery room, have passed the hospital assessment, and have been authorized the forceps midwifery technique while midwives were senior aided birth attendants with no less than 5-year midwifery experience.

### 3.1. Preoperative Verification

Prior to forceps implementation, residents reported to the senior physician about the progress of delivery, previous history, the ultrasound results in details, and the current dilemma. Then, the senior physician checked the delivery safety checklist (Table 1) and performed an abdominal examination to reestimate the fetal size and fetal lie, then clarified the fetal station, fetal head descent, and the clinical adequacy of the maternal pelvis by vaginal examination. A discussion was taken among the senior physician, residents, and midwives to determine whether the forceps assisted delivery was reasonable or not, and then an appropriate type of forceps would be selected as a result. The selection criteria of forceps were as following: Simpson forceps were selected when the fetal position was the anterior occipital position or nonoccipital position turned into anterior occipital position by hand, whereas Kielland forceps were selected when the fetal position was transverse and hand rotation fails. Then, the first part of the forceps delivery checklist (Table 2) would be completed.

### 3.2. Preoperative Preparation

After the verification work is finished, the senior physician would tell the pregnant woman and her guardian the necessity and risks of forceps delivery and then indicate them to sign an informed consent form. Meanwhile, the residents and midwives completed the preparation of the midwifery kit, forceps, and neonatal asphyxia resuscitation equipment and completed the second part of the forceps assisted delivery verification form (Table 2).

### 3.3. Intraoperative Verification

To ensure bladder empty and adequate analgesia, if necessary, a lateral perineal incision would be recommended. The senior physician should reconfirm the fetal position that was occipitoanterior by vaginal examination. If it was posterior or transverse occipital, Sb should transfer them into anterior by manually rotating the fetal head between contractions. Once the rotation failed, Kielland forceps were used to rotate the fetal head into an occipitoanterior position.

#### 3.3.1. Operations of Kielland Forceps

Placing forceps on both sides of the fetal head by the one-handed forceps method, then the operator clamped forceps after confirming no soft tissues of the birth canal were clamped. After reconfirming the correct position of the forceps, the operator took a standing position, with the index and middle fingers placed on the two shoulders of the forceps, and then pulled the forceps along the pelvic axis. In the first place, the direction of downward and outward traction was at an angle of 30 degrees below the horizontal plane. As the fetal head was gradually delivered, the handle of the forceps was slowly lifted. When the fetal head was exposed, the direction of traction was changed to horizontal, and the forceps were removed when the fetal head was pulled to the crown. The midwife continued to assist in the delivery of the fetal head and carcass.

#### 3.3.2. Operations of Simpson Forceps

Doctor's posture and the traction direction of Simpson forceps were the same as Kielland forceps, while the difference was that in an operation of Simpson forceps when the vaginal opening exposed the forehead of the fetus, the handle of the forceps was gradually lifted up to help the fetal head stretch up. When the mandible of the fetus could be touched, the forceps would be removed, and the midwife continued to deliver the fetal head and carcass.

### 3.4. Postoperative Verification

Once the baby was born, a neonatologist would conduct a careful physical examination. The examination included heart rate, respiration, muscle tension, body reflexes, skin color, facial indentation or abrasion, scalp hematoma, clavicle fracture, and organ dysplasia and then performed Apgar scores. At the same time, the umbilical artery was taken for blood gas analysis to comprehensively evaluate the condition of the newborn. Assessment criteria for neonatal asphyxia: (1) mild asphyxia: Apgar score 1 min ≤ 7, or 5 min ≤ 7, with umbilical artery blood pH < 7.2, and (2) severe asphyxia: Apgar score 1 min ≤ 3 or 5 min ≤ 5, with umbilical artery blood pH < 7.0 [16]. The birth canal was examined by obstetricians and midwives; if there was a laceration of the birth canal, it needed to be sutured. In addition, as forceps midwifery was one of the high-risk factors of postpartum hemorrhage, we gave parturient prophylactic drug treatment to promote uterine contraction immediately after shoulder delivery. If postpartum hemorrhage occurred, the cause of the hemorrhage should be identified quickly, then therapeutic drugs, surgical hemostasis, and even blood products infusion had to be carried out.

## 4. Traditional Management Scheme of Forceps Delivery

Before implementation of forceps, the delivery process and related auxiliary examination should be checked by the senior physician. After learning fetal head station, fetal position, auricle direction by vaginal examination, the senior physician decided whether it is necessary to carry out forceps delivery or not. Once forceps delivery was decided, the senior physician implemented or instructed residents to perform forceps delivery. The operations of forceps were the same as the study group.

## 5. Observed Indicators

We evaluated the clinical effects of form-based forceps management by the following indicators: success rate of primary forceps traction, rate of perineal laceration, postpartum hemorrhage, neonatal asphyxia, intracranial hemorrhage, and facial skin injury.

## 6. Statistical Analysis

The sociodemographic characteristics and delivery-related data of the subjects were collected from the electronic case system. We analyzed the skewness and kurtosis of the patients' clinical data. Normally distributed data such as age, gestational age, and body mass index (BMI) were expressed as mean ± standard deviation, nonnormally distributed data such as gravidity and parity times were expressed as median and interquartile spacing, and qualitative information was expressed as composition ratio. We used an independent sample *t*-test to analyze the potential statistical differences of age, gestational age, and BMI; used Mann–Whitney *U* test to analyze the statistical differences of gravidity, parity times, perineal laceration, and neonatal asphyxia; and used the chi-square test to compare the composition ratio between the two groups. The *P* value was two-sided and the result was considered significantly different at *P* < 0.05. All the above-mentioned analyses were carried out with SPSS software.

## 7. Results

### 7.1. Clinical Characteristics

During the period from January 1, 2017, to December 31, 2018, the number of forceps deliveries in our hospital was 626, while the number of cumulative deliveries and vaginal deliveries was 44,601 and 24,593, respectively. From January 1, 2019, to December 31, 2020, there were 634 forceps deliveries, 42,409 total deliveries and 23,398 vaginal deliveries. The details are shown in Table 3. Summary statistics of clinical data of the two study groups are shown in Table 4, indicating little statistical difference between the two groups.

### 7.2. Maternal Outcomes

The success rate of forceps delivery was 100% in both groups. All of the pregnant women in the study group had successful one-time traction, while three patients in the control group had failed in their first traction. The main reason for failure was inaccurate forceps placement due to fetal position error, and we had second-time successful traction after repositioning forceps. Comparing the success rate of disposable forceps traction between the two groups, it was found that the success rate of the study group was slightly higher than that of the control group, although the differences were not obvious. The rates of postpartum hemorrhage, second-degree perineal laceration, and third-degree perineal laceration in the study group were 16.09%, 11.99%, and 0.32%, while those in the control group were 24.92%, 18.85%, and 0.48%. It was found that the rate of postpartum hemorrhage and the degree of perineal laceration in the study group were significantly lower than those in the control group (Table 5).

### 7.3. Neonatal Outcomes

There were 634 newborns in the study group, of which 7 had mild asphyxia and no severe asphyxia. There were 626 newborns in the control group, including 13 cases of mild asphyxia and 1 case of severe asphyxia due to intracranial hemorrhage. There was no significant difference in neonatal asphyxia between the two groups. In the control group, one newborn had intracranial hemorrhage. It was a case of a second traction after repositioning the forceps due to incorrect judgment of fetal position, and the possible cause of intracranial hemorrhage was considered to be excessive compression of the fetal head. In addition, the incidence of facial skin injury was 3.94% in the study group, which was significantly lower than that in the control group (Table 6).

## 8. Discussion

Since 1996, with the improvement of cesarean delivery techniques and the enhancement of pregnant women's awareness of safe delivery, the rate of cesarean section has increased in both developed and developing countries, far exceeding the alert level of the cesarean delivery rate set by the World Health Organization [17, 18]. However, the cesarean section was not as safe as that we thought. In 2007, a study from Canada showed that the risk of serious maternal illness was three times higher in patients who had a cesarean section than in those who had a vaginal delivery [19]. In the last 2 years, a number of studies had shown that unnecessary cesarean sections increased maternal and neonatal risks, even if it might increase maternal mortality [15, 20]. In short, we believed that vaginal delivery was the safer and more cost-effective way of delivery.

During the second stage of labor, when vaginal delivery became difficult, forceps delivery was an important measure to solve cephalic dystocia. Studies had shown that low forceps or export forceps performed by experienced and trained doctors in the second stage of labor might safely reduce risks of cesarean delivery and that vaginal surgical delivery should be considered a safe and acceptable alternative to cesarean delivery [12, 15, 21, 22]. However, forceps delivery is highly required for obstetricians and midwifery; if performed improperly, it might cause serious birth canal injuries, postpartum hemorrhage, neonatal birth injuries, and other complications. All of the complications would do great harm to the mother and the infant. Therefore, we needed to try to avoid complications.

In 2016, our hospital improved the traction method of Kielland forceps which was consistent with the previously described method. We found that the improved method could reduce the complications of forceps delivery to some extent, but because these technical improvements relied more on the experience of operating physician, and a study showed that the probability of error in judgment of fetal position and parameters was 50%–80% for residents and 36%–80% for attending physicians [23]; therefore, the results we achieved were not significant. Subsequently, we carefully analyzed cases of forceps delivery that had serious complications before January 1, 2019, and we found the main causes of these complications were incomplete preoperative assessment and improper operation, while the underlying cause was the lack of standardized management of forceps delivery.

The workload of the medical staff in the delivery room was heavy. In order to remind the medical staff to pay attention to the surgical risk of each forceps delivery, we have changed our traditional forceps delivery protocol and have been using a form-based forceps delivery management protocol since 2019. We established a delivery safety checklist and a forceps delivery checklist. The forceps delivery checklist was described in detail according to four parts: preoperative verification, preoperative preparation, intraoperative operation, and postoperative examination. Specific verification requirements were put forward from aspects of prerequisites of forceps midwifery, communication, personnel and facility preparation, key points of operation, examination of postoperative maternal and fetal complications, and so forth. At the same time, senior physicians, residents, and midwives were required to participate in and independently verify the key steps in the process. In this way, we could avoid not only the omission of preparations by medical staff due to fatigue, negligence, and emergency but also incorrect operations due to inexperience and so forth. In addition, we also emphasized the postoperative verification of maternal and infant complications, identified the causes of complications timely, and correct errors early. Through this way, we could improve the forceps delivery continuously.

After carefully checked by senior doctors, residents, and midwives before the forceps delivery, the fetal positions of all fetuses among 634 patients in the study group were accurately judged and the traction was successful at one time. While verification of fetus in the control group only relied on senior doctors, there were three failed tractions at one time due to errors in judging the fetal position, caused by negligence, fatigue, or tension of senior doctors. Also, the incidence of perineal lacerations, postpartum hemorrhage, and neonatal facial skin damage were significantly lower than those of the control group based on no difference in neonatal asphyxia. All of these fully demonstrated that the form-based management of forceps delivery could strengthen cooperation between doctors and midwives, help medical staff comprehensively, and accurately evaluate the necessity and operating conditions of the forceps delivery, aimed to avoid errors in forceps operation and reduce complications of mother and neonates. At the same time, it might not delay the delivery of high-risk newborns. Therefore, form-based management of forceps delivery was beneficial to obstetric forceps management.

## 9. Conclusions

In this paper, we found that the form-based forceps delivery management could improve the success rate of the one-time forceps traction scheme and reduce the maternal rate of postpartum hemorrhage and risk of perineal laceration under the intelligent medical model. In the context of promoting vaginal delivery and reducing the rate of first cesarean delivery, we need to improve the skill level of obstetric medical staff in forceps delivery and strengthen the management of forceps delivery in the department and homogenize the forceps delivery.

## Figures and Tables

**Table 1:** The delivery room safe delivery verification form.

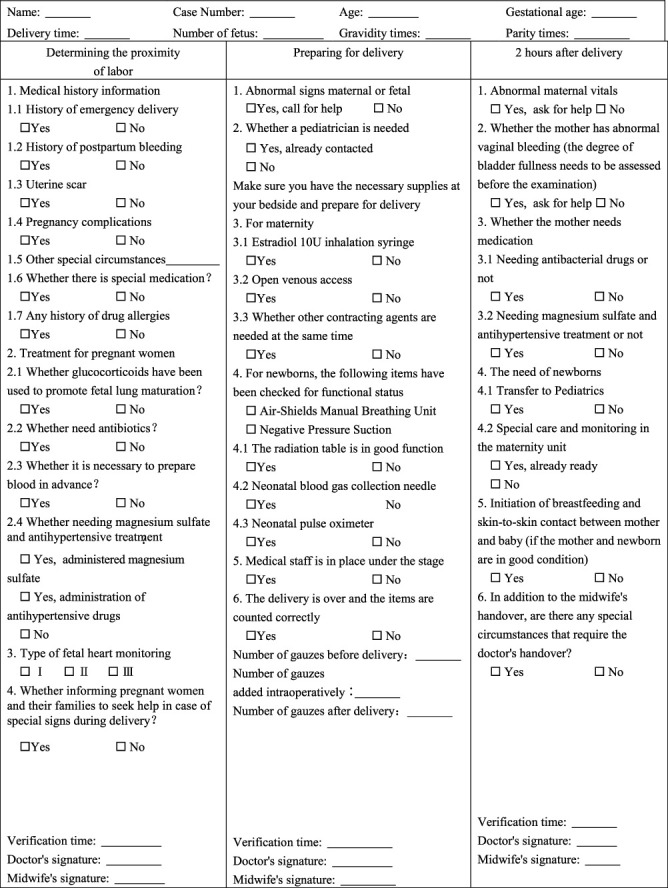

**Table 2:** The forceps delivery verification form.

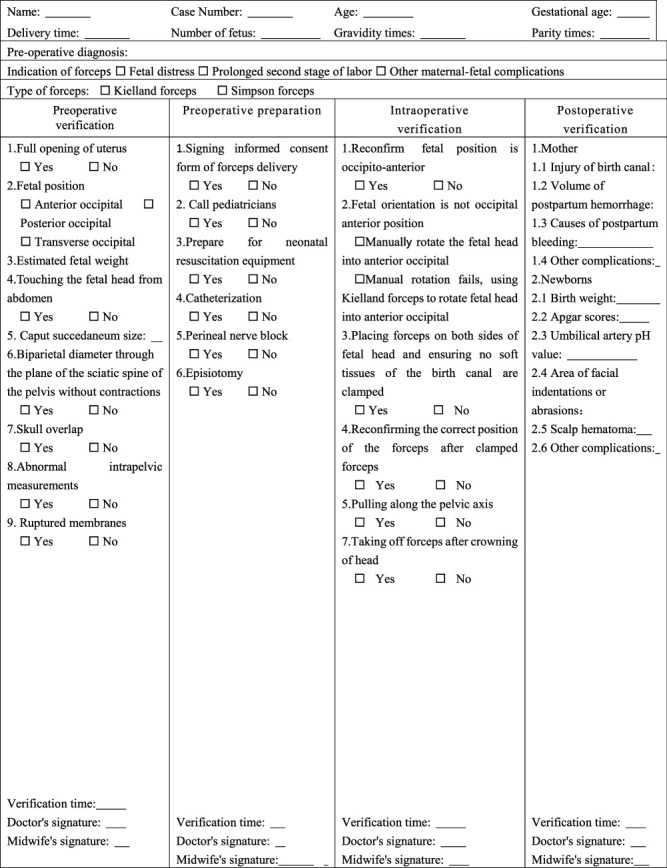

**Table 3:** The status of vaginal deliveries and forceps deliveries during the last 4 years.

	01/01/2017–31/12/2018	01/01/2019–31/12/2020	*P* value	Method
Total deliveries (case)	44601	42409		
Vaginal deliveries (case/rate)	24593 (55.14%)	23398 (55.17%)	0.924	Pearson
Forceps deliveries (case/rate)	626 (2.55%)	634 (2.71%)	0.261	Pearson

**Table 4:** Descriptive statistics of basic information of study population.

Features	Study group (*n* = 634)	Control group (*n* = 626)	*P* value	Method
Age (years)	29.33 ± 5.22	28.40 ± 5.72	0.697	Independent sample *t*
Gestational age (days)	273.29 ± 16.01	270.00 ± 15.03	0.149	Independent sample *t*
Gravidity (times)	2.00 (1.00–3.00)	3.00 (1.50–5.00)	0.28	Mann–Whitney
Parity (times)	0.50 (0.00–1.00)	0.50 (0.00–1.50)	0.71	Mann–Whitney
BMI (kg/m^2^)	25.79 ± 3.36	25.74 ± 2.03	0.973	Independent sample *t*

Data are presented as mean ± standard deviation or median (interquartile spacing). BMI: body mass index.

**Table 5:** Maternal outcomes of study population.

	Study group (*n* = 634)	Control group (*n* = 626)	*P* value	Method
Successful one-time traction (case/rate)	634 (100%)	623 (99.52%)	0.122	Fisher's exact
Postpartum hemorrhage (case/rate)	102 (16.09%)	156 (24.92%)	<0.001	Pearson

Perineal laceration				
Second degree (case/rate)	76 (11.99%)	118 (18.85%)	0.001	Mann–Whitney
Third degree (case/rate)	2 (0.32%)	3 (0.48%)		

**Table 6:** Newborn outcomes of study population.

	Study group (*n* = 634)	Control group (*n* = 626)	*P* value	Method
Neonatal asphyxia				
Mild asphyxia (case/rate)	7 (1.10%)	13 (2.08%)	0.116	Mann–Whitney
Severe asphyxia (case/rate)	0 (0)	1 (0.16%)		
Intracranial hemorrhage (case/rate)	0 (0)	1 (0.16%)	0.497	Fisher's exact
Facial skin injury (case/rate)	25 (3.94%)	82 (13.10%)	<0.001	Pearson

## Data Availability

The datasets used and/or analyzed during the current study are available from the corresponding author on reasonable request.
